# Mitochondria-Targeting and Oxygen Self-Supplying Eccentric Hollow Nanoplatform for Enhanced Breast Cancer Photodynamic Therapy

**DOI:** 10.1155/2024/6618388

**Published:** 2024-02-01

**Authors:** Jing Li, Yu Wang, Jun Tao, Xiaodan Su, Feipeng Zhu, Wei Lu, Xiaolin Han, Meng Dang, Lixing Weng

**Affiliations:** ^1^Key Laboratory for Organic Electronics & Information Displays and Jiangsu Key Laboratory for Biosensors, Institute of Advanced Materials, Jiangsu National Synergetic Innovation Centre for Advanced Materials, Nanjing University of Posts and Telecommunications, Nanjing 210023, China; ^2^Department of Radiology, The First Affiliated Hospital of Nanjing Medical University, Nanjing 210029, China; ^3^State Key Laboratory for Modification of Chemical Fibers and Polymer Materials, College of Materials Science and Engineering, Institute of Functional Materials, Donghua University, Shanghai 201620, China; ^4^College of Geography and Biological Information, Nanjing University of Posts and Telecommunications, Nanjing 210046, China

## Abstract

Photodynamic therapy (PDT) has received increasing attention for tumor therapy due to its minimal invasiveness and spatiotemporal selectivity. However, the poor targeting of photosensitizer and hypoxia of the tumor microenvironment limit the PDT efficacy. Herein, eccentric hollow mesoporous organic silica nanoparticles (EHMONs) are prepared by anisotropic encapsulation and hydrothermal etching for constructing PDT nanoplatforms with targeting and hypoxia-alleviating properties. The prepared EHMONs possess a unique eccentric hollow structure, a uniform size (300 nm), a large cavity, and ordered mesoporous channels (2.3 nm). The EHMONs are modified with the mitochondria-targeting molecule triphenylphosphine (CTPP) and photosensitizers chlorin e6 (Ce6). Oxygen-carrying compound perfluorocarbons (PFCs) are further loaded in the internal cavity of EHMONs. Hemolytic assays and *in vitro* toxicity experiments show that the EHMONs-Ce6-CTPP possesses very good biocompatibility and can target mitochondria of triple-negative breast cancer, thus increasing the accumulation of photosensitizers Ce6 at mitochondria after entering cancer cells. The EHMONs-Ce6-CTPP@PFCs with oxygen-carrying ability can alleviate hypoxia after entering in the cancer cell. Phantom and cellular experiments show that the EHMONs-Ce6-CTPP@PFCs produce more singlet oxygen reactive oxygen species (ROSs). Thus, in vitro and in vivo experiments demonstrated that the EHMONs-Ce6-CTPP@PFCs showed excellent treatment effects for triple-negative breast cancer. This research provides a new method for a targeting and oxygen-carrying nanoplatform for enhancing PDF effectiveness.

## 1. Introduction

Photodynamic therapy (PDT) has attracted widespread attention for cancer therapy, in which the photosensitizers (PSs) effectively kill cancer cells *via* converting O_2_ into reactive oxygen species (ROSs) under the irradiation of a laser [1]. PDT shows great promise because of its advantages of low trauma, high spatial selectivity, and a short course of treatment [2–6]. Two crucial issues should be considered for improving the PDT effectiveness of cancer. First, oxygen plays a significant role in the production of ROSs during PDT [7–11]. However, aggressive proliferation of solid cancer and the twisted vascular system lead to liquefaction necrosis [12], resulting in a partial pressure of oxygen below 5 mmHg [13]. Second, the lifetime and diffusion distance of ROSs are as short as 200 ns and 20 nm, respectively, resulting in the ROSs only destroying the nearby molecules [14–17]. Therefore, the targeting of PSs in organelles is particularly important for improving the therapeutic efficacy of cancer [18–21].

Mesoporous organosilica nanoparticles (MONs) have been widely used for the diagnosis and treatment of tumor due to their high specific surface area (>800 m^2^/g), homogeneous mesoporous channels, easy surface modification, and good biocompatibility [22–27]. We speculate that loading oxygen-carrying molecules into mesoporous organosilica is a feasible strategy to increase oxygen content in cancer and alleviate cancer hypoxia [28–30]. In addition, mitochondria is the main energy production center and the site of aerobic respiration. Mitochondria plays an important role in the pathway of ROS-induced apoptosis [31–33]. Yue et al.'s group developed various mitochondria-targeting drug delivery systems based on the triphenylphosphonium (CTPP) molecular structure and achieved excellent targeted therapeutic effects for cancer therapy [34–36]. Therefore, developing a photosensitizer carrier that can both deliver oxygen to relieve hypoxia and modify CTPP molecules to achieve targeted delivery to mitochondria is expected to enhance the therapeutic effectiveness of PDT.

Herein, we fabricated a mitochondria-targeting and oxygen-carrying nanoplatform for enhancing the PDT effect. Typically, eccentric hollow mesoporous organicsilica nanoparticles (EHMONs) were synthesized by anisotropic encapsulation and hydrothermal etching [37, 38]. The EHMONs possessed a unique eccentric hollow structure, uniform size, large cavity (150 nm), and ordered mesoporous channel (2.3 nm). The nanoparticles were functionalized by mitochondrial targeting molecules CTPP and photosensitizers Ce6, and oxygen-carrying point perfluorocarbons (PFCs) were loaded in the internal cavity. The prepared EHMONs-CTPP-Ce6@PFCs possessed good biocompatibility and exhibited higher singlet oxygen generation efficiency. Cell toxicity tests showed that the EHMONs-CTPP-Ce6@PFCs can effectively kill 4T1 triple-negative breast cancer cells.

## 2. Methods

### 2.1. Materials

Tetraethyl orthosilicate (TEOS, 28.4%), concentrated hydrochloric acid (HCl, 37 wt%), cetyltrimethylammonium bromide (CTAB, 99%), ammonia aqueous solution (NH_3_·H_2_O, 25 wt%), (3-aminopropyl) triethoxysilane (APTES), and anhydrous ethanol were purchased from Sinopharm ChemicalReagent Co., (Shanghai, China). 1,2-Bis (triethoxysilyl)ethane (BTSE) was acquired from Sigma-Aldrich Co., Ltd. (Shanghai, China). Phosphate-buffered saline (PBS), fetal bovine serum (FBS), cell counting kit-8 (CCK-8), and Roswell Park Memorial Institute (RPMI) 1640 medium were purchased from Nanjing Keygen Biotech Co., Ltd. (Nanjing, China). N-(3-dimethylaminopropyl)-N′-ethylcarbodiimide hydrochloride (EDC), N-hvdroxysulfosuccinimide (NHS), and triphenylphosphine (CTPP) were purchased from Shanghai Aladdin Biochemical Technology Co., Ltd. (Shanghai, China). 4,6-Diamino-2-phenylindole (DAPI) was purchased from Santa Cruz Biotechnology (Santa Cruz, USA). Singlet oxygen sensor green (SOSG) and 2′,7′-dichlorofluorescin diacetate (DCFH-DA) were purchased from Thermo Fisher Technology Co., Ltd. (Shanghai, China). 4T1 human breast cancer cells were acquired from the American Standard Biological Products Collection Center. (Beijing, China).

### 2.2. Synthesis

Typically, 75 mL of ethanol was added to 170 mL of CTAB (6 mM) in water. Then, 0.1 mL of ammonia was added to the mixed solution. The solution was stirred at 35°C and 500 rpm. After 5 min, 0.2 mL of tetraethyl orthosilicate (TEOS) was slowly added, and the temperature of the reaction system was raised to 60°C. After 24 h, mesoporous silica nanoparticles (MSNs) were obtained and washed three times with ethanol. Next, 3.5 mg of MSNs was dispersed in 0.5 mL of ethanol, 8.5 mL of deionized water, 15 mg of CTAB, and 0.7 mL of ammonia-mixed solution, and the solution was stirred evenly at 35°C and 500 rpm for 30 min. Then, 0.1 mL of 1,2-bis (triethoxysilane) ethane (BTSE) was slowly added to the mixed solution, and the solution was stirred for 3 h. The white flocculent precipitate was washed with ethanol three times and water once. The precipitate was dispersed in 35 mL of water for 12 h at 80°C. The EHMONs were collected *via* centrifugation and redispersed in an ethanol/HCl solution (Vethanol = 10 mL, VHCl = 20 *μ*L) to remove CTAB.

One milliliter of CTPP (20 mg/mL) and 1 mL of photosensitizer Ce6 (20 mg/mL) were mixed with 0.5 mL of EDC (20 mg/mL, dissolved in DMF) and 0.5 mL of NHS (20 mg/mL, dissolved in DMF), respectively. The mixture was shaken at room temperature for 3 h to activate the carboxyl groups. EHMONs were dispersed in 42 mL of ethanol, and 0.2 mL of ammonia and 0.3 mL of APTES were added to the solution. The mixed solution was shaken for 12 h. The precipitate was collected by centrifugation. 30 mg of the precipitation was dispersed in the mixed solution of activated CTPP and Ce6. After 12 h, the surface-functionalized EHMONs-Ce6-CTPP were collected by centrifugation.

### 2.3. Loading Oxygen

At normal temperature and pressure, a large amount of oxygen can be directly dissolved, and its oxygen solubility is three times that of blood (35–70 ml/dl at 25 degrees Celsius). Therefore, 1 mg of EHMONs-Ce6-CTPP was centrifuged and washed twice. Under vacuum, 0.1 mL of perfluoropentane solution was quickly added to EHMONs-Ce6-CTPP, and ultrasound was performed in an ice bath with a temperature below 4°C for 2 min to obtain EHMONs-Ce6-CTPP@PFC.

### 2.4. Hemolytic Assay and *In Vitro* Toxicity

In order to explore the hemolysis performance of EHMONs, one milliliter of blood obtained from Jinling Hospital was centrifuged at 2000 rpm for 5 min to collect the red blood cells (RBCs). The obtained RBCs were diluted with physiological saline to 2 ml. The RBCs suspension (0.2 ml) was incubated with the EHMONs-Ce6-CTPP in physiological saline (0.8 ml) at 35°C for 2 h. RBCs incubated with water and physiological saline were set as the positive and negative control, respectively. Finally, the supernatants were collected to measure the absorbance at 630 nm. The hemolysis percentage of RBCs (%) was calculated by the following formula:

(1) $$\begin{array}{c} \text{Hemolysis rate}=\frac{\text{absorbance of sample }–\text{ absorbance of}\;\text{negative control}}{\text{absorbance of positive control}?\text{absorbance of}\;\text{negative control}}\times 100\%. \end{array}$$

To determine the *in vitro* cytotoxicity, 4T1 human breast cancer cells were cultured in RPMI 1640 medium containing 10% fetal bovine serum and 1% penicillin-streptomycin at 5% CO_2_ concentration and 95% relative humidity, then seeded in 96-well plates (1 × 10^4^ cells/per well) for 24 h. Afterwards, different concentrations of EHMONs-Ce6-CTPP were dispersed in RPMI 1640 medium (100 *μ*L) and added to each well. After 24 h or 48 h, the medium from each well was replaced with 100 *μ*L complete medium containing 10 *μ*L CCK-8. After 1 h, the absorbance of the medium in each well was measured at 490 nm with a microplate reader. Cell activity was calculated by the following formula:

(2) $$\begin{array}{c} \text{Cell viability}=\frac{\text{the absorbance at }490\text{ nm of experimental group}}{\text{the absorbance at }490\text{ nm of control group}}\times 100\%. \end{array}$$

### 2.5. Targeted Mitochondria

To detect performance of EHMONs in mitochondrial targeting, 4T1 human breast cancer cells were incubated in the culture plate at an inoculation density of 1 × 10^3^. 100 *μ*L Ce6, EHMONs-Ce6-CTPP, and EHMONs-Ce6-CTPP@PFC medium suspension (with Ce6 equivalent concentration of 1 *μ*M) were then added to the cells, respectively. After 2 h, the culture medium was sucked out, and the cells were washed three times with PBS to remove the residual culture solution. The cells were stained with mitochondrial stain for 20 min. Then, excess stain was sucked out, and the cells were washed with PBS three times. DAPI dye was added to the cells to stain the nuclei. After 10 min, the staining solution was sucked out, and 100 *μ*L PBS was added to the cells. The cells were observed using laser confocal microscope (CLSM). The fluorescence localization results were statistically analyzed by ImageJ software.

### 2.6. *In Vitro* Singlet Oxygen Detection

To detect the singlet oxygen generation ability of EHMONs *in vitro*, 100 *μ*L Ce6, EHMONs-Ce6-CTPP, and EHMONs-Ce6-CTPP@PFC medium suspension with equivalent Ce6 concentration (1 *μ*M) were mixed with 10 *μ*L SOSG (50 *μ*M) and added to the cells, respectively. The cells were irradiated by a 660 nm laser with a power of 1 W/cm^2^ for 0, 1, 3, 5, and 10 min, respectively. Each experimental group was set up in three wells. The fluorescence emission intensity of the sample well was measured using a microplate reader at the wavelength of 530 nm.

To further detect the generation of singlet oxygen at the cellular level, 4T1 human breast cancer cells were incubated for 24 h, 100 *μ*L of Ce6, EHMONs-Ce6-CTPP, and EHMONs-Ce6-CTPP@PFC medium suspension with equivalent Ce6 concentration (1 *μ*M) were added to the cells, respectively. Then, the cells were incubated in the dark for 12 h, the medium was removed from the well, and the cells were washed three times with PBS. Then, 100 *μ*L of DCFH-DA (20 *μ*M) was added to the cells. After 4 h, the residual solution was sucked out, and the cells were washed once. Then, 100 *μ*L of 1640 medium was added to the cells, and each well was irradiated with a 660 nm laser with a power of 1 W/cm^2^ for 5 min. The fluorescence emission intensity of each well was recorded with a microplate reader at 485 nm.

### 2.7. *In Vitro* Photodynamic Therapy

To evaluate the effect of photodynamic therapy on EHMONs, 4T1 human breast cancer cells were incubated for 24 h, and then 100 *μ*L Ce6, EHMONs-Ce6-CTPP, and EHMONs-Ce6-CTPP@PFC medium suspension with equivalent Ce6 concentration (1 *μ*M) were added to the cells, respectively. After 12 h, the cells were washed three times with PBS and 100 *μ*L of fresh medium was added to the cells, and they were irradiated with a 660 nm laser for 5 min (1 W/cm^2^). The medium in each well was replaced with 100 *μ*L medium containing 10 *μ*L CCK-8. After 1 h, the absorbance value of each well at 490 nm was detected by using a microplate reader, and 5 multiple wells were set for each experimental group. 4T1 breast cancer cell activity was calculated by the following formula:

(3) $$\begin{array}{c} \text{Cell viability}\left(100\%\right)=\frac{\text{the absorbance at }490\text{ nm of experimental group}}{\text{the absorbance at }490\text{ nm of control group}}\times 100\%. \end{array}$$

### 2.8. *In Vivo* Antitumor Effect

4T1 breast tumor model was constructed by injected into 1 × 10^6^ cells into BALB/c mice on day 0. When the tumors reached to ∼130 mm^3^, the mice were randomly divided into 4 groups and injected with PBS, Ce6, EHMONs-Ce6@PFC, and EHMONs-Ce6-CTPP@PFC at an equivalent Ce6 dose of 20 *μ*g per mouse at days 7, followed by 635 nm irradiation with a 660 nm laser for 5 min (1 W/cm^2^) on day 8. Tumor volumes and body weights were recorded every two days during the two weeks. The tumor volume was calculated according to the formula: tumor volume = length × width^2^ × 0.5 mm^3^.

### 2.9. Characterization

S4800 scanning electron microscope (SEM, Hitachi, Tokyo), HT7700 transmission electron microscope (TEM, Hitachi, Tokyo, Japan), and FEI Talos F200x electron microscope were employed to capture SEM, TEM, and elemental mapping images, respectively. Nitrogen adsorption-desorption isotherms were measured using a Micromeritics ASAP 2020 analyzer at −196°C. Before the measurements, the samples were degassed under vacuum at 150°C for at least 10 h. The specific surface area (*S*_BET_) was calculated with the Brunauer–Emmett–Teller (BET) method using the adsorption data in a relative pressure (*P/P*_0_) range from 0.14 to 0.25. The pore size distribution was obtained by applying proper nonlocal density functional theory (NLDFT) methods from the adsorption branch of isotherms. A Brookhaven Zeta-PLAS analyzer (USA) was employed to characterize the hydrodynamic size and surface electronegativity of the nanoparticles. An Infinite M200 PRO microplate reader (Tecan, Switzerland) was employed to measure the absorbance. Material characterization was repeated at least three times.

## 3. Results and Discussion

**Figure 1:**
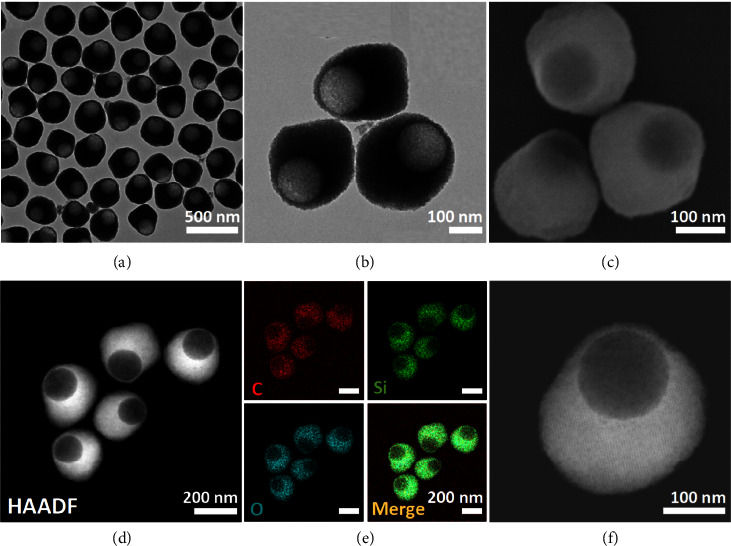
(a, b) TEM, (c) SEM, (d) STEM-HADDF, (e) elemental mapping, and (f) high-magnification TEM images of the EHMONs.

Eccentric hollow mesoporous organosilica nanoparticles with organic-inorganic hybrid frameworks were prepared by anisotropic encapsulation and hydrothermal etching. In particular, MSNs with a uniform particle size were used as a seed. Mesoporous organosilica was anisotropically encapsulated on the MSNs to form the eccentric mesoporous organosilica nanoparticles by using 1,2-bis (triethoxysilyl)ethane (BTSE) as an organosilica precursor and the hexadecyltrimethylammonium bromide (CTAB) as a structural template. After hydrothermal treatment, the MSNs were etched away, and eccentric hollow mesoporous organosilica nanoparticles (EHMONs) were formed. TEM images showed that the obtained EHMONs had very good dispersion and uniform size (300 nm) (Figure 1(a)). High-magnification TEM images showed clearly that the EHMONs possessed a large internal cavity (about 140 nm) which was conducive to the storage of guest molecules (Figure 1(b)). SEM and high-angle annular dark-field scanning TEM (STEM-HAADF) images revealed that the EHMONs have ultrathin shells near the cavity side with a size of about 16 nm (Figures 1(c) and 1(d)). The ultrathin shell was extremely favorable for the diffusion of drug molecules. Elemental mapping images clearly show that C, Si, and O elements were evenly distributed in the EHMONs (Figure 1(e)), indicating their organic-inorganic hybrid frameworks. High-magnification TEM images showed that the surface of EHMONs possesses a highly ordered mesoporous structure (Figure 1(f)), which extremely facilitates the loading and release of drug molecule.

**Figure 2:**
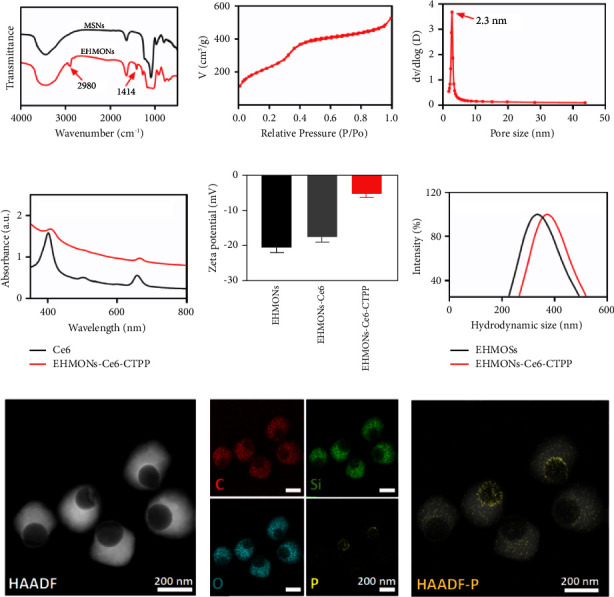
(a) FT-IR images of MSNs and EHMONs. (b) Isotherm curve of nitrogen adsorption and desorption. (c) Pore size distribution curve. (d) Ultraviolet absorption spectra of the EHMONs-Ce6-CTPP. (e) Hydration particle size of MSNs, EHMONs, and EHMONs-Ce6-CTPP. (f) Zeta potentials of EHMONs, EHMONs-Ce6, and EHMONs-Ce6-CTPP. (g–i) Element mapping images of EHMONs-Ce6-CTPP.

We further investigated the physicochemical properties of the EHMONs. The Fourier transform infrared spectrum (FT-IR) of the EHMONs showed two characteristic peaks at 2980 cm^−1^ and 1414 cm^−1^ (Figure 2(a)), which were assigned to the C-H stretching vibration. In contrast, the peaks were absent in MSN, indicating that EHMONs possessed the organic-inorganic hybrid framework. Nitrogen adsorption-desorption isotherms of the EHMONs showed a type IV curve (Figure 2(b)), indicating a typical mesoporous structure. In addition, the pore size of the mesoporous shell was calculated to be 2.3 nm by nonlocal density functional theory (Figure 2(c)). UV-visible spectra of the EHMONs-Ce6-CTPP showed two absorption peaks at 400 nm and 660 nm (Figure 2(d)), which coincided with the characteristic absorption peak of Ce6, indicating successful modification of Ce6. The loading content of Ce6 in the EHMONs-Ce6-CTPP was determined to be up to 30 *μ*g/mg. The zeta potential of the EHMONs, EHMONs-Ce6, and EHMONs-Ce6-CTPP was measured to be −21 mV, −17.5 mV, and −5 mV (Figure 2(e)), respectively, indicating the successful modification of functional molecules. Dynamic light scattering (DLS) showed the hydrodynamic diameters of EHMONs and EHMONs-Ce6-CTPP were 370 nm and 390 nm, respectively. The polydispersibility index (PDI) values of the EHMONs and EHMONs-Ce6-CTPP were 0.118 and 0.123, respectively, demonstrating excellent uniformity (Figure 2(f)). STEM-HAADF image of the modified nanoparticles clearly showed no change in the morphology (Figure 2(g)). Elemental mapping images showed that C, O, Si, and P elements were distributed in the organic-inorganic hybrid framework of the EHMONs-Ce6-CTPP (Figure 2(h)), indicating the successful modification of CTPP on the nanoparticles.

**Figure 3:**
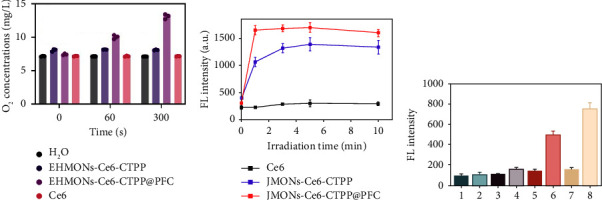
(a) The oxygen concentrations in water after adding H_2_O, EHMONs-Ce6-CTPP, EHMONs-Ce6-CTPP@PFC, and Ce6. (b) The ROSs produced by the Ce6, EHMONs-Ce6-CTPP, and EHMONs-Ce6-CTPP@PFC under different laser irradiation times. (c) Diagram of intracellular ROSs produced by different materials (1: control, 2: control + laser, 3: Ce6, 4: Ce6 + laser, 5: EHMONs-Ce6-CTPP, 6: EHMONs-Ce6-CTPP + laser, 7: EHMONs-Ce6-CTPP@PFC, 8: EHMONs-Ce6-CTPP@PFC + laser, power: 1 W/cm^2^).

The PFC possessed the ability to carry oxygen to relieve the hypoxic microenvironments, so, the oxygen concentrations in H_2_O, EHMONs-Ce6-CTPP, EHMONs-Ce6-CTPP@PFC, or Ce6 solution was detected. The result showed that the oxygen concentrations in the EHMONs-Ce6-CTPP@PFC solution increased to 13.4 mg/L within 2 min and remained constant for over 5 min, which is 1.9, 1.7, and 1.8-fold higher than that in H_2_O, EHMONs-Ce6-CTPP, and Ce6 groups, respectively (Figure 3(a)). Furthermore, the singlet oxygen generation capacity of EHMONs-Ce6-CTPP@PFC was also studied. SOSG was used to detect the generation of singlet oxygen. As shown in Figure 3(b), the fluorescence signal intensity of SOSG in the Ce6 group did not change significantly with the increase in laser irradiation time. In contrast, the fluorescence signal intensity of the EHMONs-Ce6-CTPP@PFC and EHMONs-Ce6-CTPP groups increased rapidly within 1 min, and tended to be stable after 5 min. Notably, the fluorescence signal intensity was much higher in the EHMONs-Ce6-CTPP@PFC group compared to other groups, indicating that the loading oxygen-carrying PFCs in EHMONs-Ce6-CTPP@PFC can significantly enhance the generation rate and amount of the singlet oxygen under laser irradiation. Furthermore, the ROSs generated at cellular level was detected using DCFH-DA. The results showed that the fluorescence signal intensities of the EHMONs-Ce6-CTPP and EHMONs-Ce6-CTPP@PFC groups were significantly higher than those of other groups after laser irradiation (Figure 3(c)). It was noteworthy that the EHMONs-Ce6-CTPP@PFC + laser showed the strongest fluorescent signal in cancer cells, suggesting an increase in ROS content.

**Figure 4:**
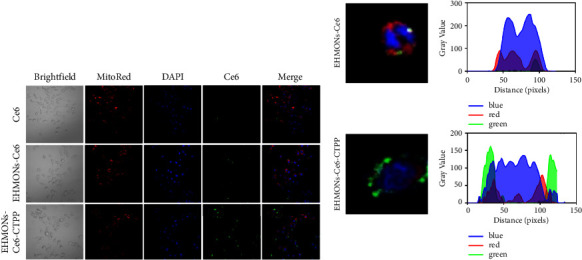
(a) Fluorescence confocal microscopy images of Ce6, EHMONs-Ce6, and EHMONs-Ce6-CTPP incubated with 4T1 cells for 2 h. (b) Analysis of images of different fluorescence localization in cells. Red, blue, and green colors indicate mitochondria and the nucleus, Ce6, respectively (analysis software: ImageJ).

The mitochondrial targeting performance of the EHMONs-Ce6-CTPP was further investigated. Compared with the other two groups, the Ce6 group showed the weakest fluorescence signal (Figure 4(a)). The fluorescence intensity of the EHMONs-Ce6 group was slightly stronger than that of the free Ce6 group. In contrast, the EHMONs-Ce6-CTPP group shows the strongest fluorescence intensity, indicating that the mitochondria-targeting molecular CTPP contributed to improving the uptake efficiency and quantity of particles by cells. The ImageJ software results showed that the position of the green fluorescence of Ce6 in the EHMONs-Ce6-CTPP group was overlapping with that of the red fluorescence of mitochondria (Figure 4(b)), and the green fluorescence intensity was significantly higher than that of the EHMONs-Ce6 group (green area). These results indicated that EHMONs-Ce6-CTPP can target mitochondria and increase aggregation in mitochondria after entering cells.

**Figure 5:**
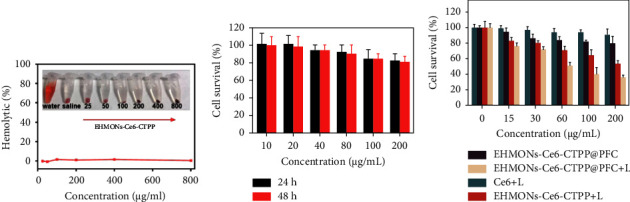
(a) Hemolytic activity of different concentrations of EHMONs-Ce6-CTPP; (b) cell survival of 4T1 cells incubated with different concentrations of EHMONs-Ce6-CTPP for 24 h and 48 h, respectively; (c) cell survival of 4T1 cells underwent treatment of Ce6 + laser, EHMONs-Ce6-CTPP@PFC, EHMONs-Ce6-CTPP + laser, and EHMONs-Ce6-CTPP@PFC + laser groups.

The hemolysis and cytotoxicity of the EHMONs were further investigated to demonstrate their potential for biomedical applications. After mixing red blood cells (RBCs) with the EHMONs-Ce6-CTPP at concentrations ranging from 25 to 800 *μ*g/mL, only slight red color was observed in the supernatants (inset in Figure 5(a)). Quantitative analysis indicated that the hemolytic activity of the EHMONs-Ce6-CTPP was 1.75% at the concentration up to 800 *μ*g/mL (Figure 5(a)), suggesting that the EHMONs-Ce6-CTPP induced very light hemolysis toward RBCs. The survival rate of 4T1 cells remained above 80% after the cells were incubated with different concentrations of EHMONs-Ce6-CTPP (less than 200 *μ*g/mL) for 24 h (Figure 5(b)). Upon further incubation to 48 h, the survival rate of 4T1 cells remained above 80%, indicating that EHMONs-Ce6-CTPP nanoparticles had good *in vitro* biocompatibility. Finally, we evaluated the photodynamic therapy effect of EHMONs-Ce6-CTPP@PFC on tumor cells. The results showed that the cell survival rate of Ce6 + laser, EHMONs-Ce6 + laser, EHMONs-Ce6-CTPP + laser, and EHMONs-Ce6-CTPP@PFC + laser groups were 90%, 79%, 53%, and 35% at a concentration of 200 *μ*g/mL, respectively, indicating that the modified targeting molecule CTPP significantly improves the effectiveness of photodynamic therapy. Notably, the EHMONs-Ce6-CTPP@PFC with oxygen-carrying PFC molecules killed about 65% of cancer cells, suggesting that the strategy of alleviating hypoxia and mitochondria-targeting effectively enhances the effect of PDT (Figure 5(c)).

**Figure 6:**
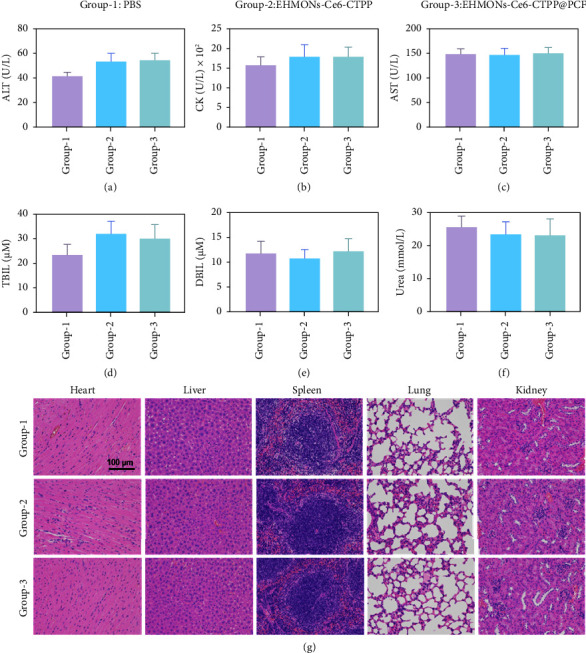
The biochemical analysis of the serum of mice treated with EHMONs-Ce6-CTPP@PFCs and PBS. *n* = 3: (a) ALT; (b) CK; (c) AST; (d) TBIL; (e) DBIL; (f) urea. (g) The assessment of main organs by H&E staining. Group-1: PBS, Group-2: EHMONs-Ce6-TCPP, Group-3: EHMONs-Ce6-TCPP@PCF.

To evaluate the safety of HMONs-Ce6-CTPP@PFCs in vivo, we investigated the blood physiological and biochemical parameters in mice treated with HMONs-Ce6-CTPP@PFCs and PBS. The results showed no significant differences in alanine aminotransferase (ALT), creatine kinase (CK), aspartate aminotransferase (AST), total bilirubin (TBIL), or direct bilirubin (DBIL) between the various groups of mice for different treatments (Figures 6(a)–6(f)). Moreover, we further examined the major organs of the mice using hematoxylin and eosin (H&E) staining. The results revealed no significant morphological changes in the major tissues and organs of mice treated with HMONs-Ce6-CTPP@PFCs compared with those treated with PBS (Figure 6(g)). These findings indicate that EHMONs-Ce6-CTPP@PFCs are safe for use in vivo during cancer treatment.

**Figure 7:**
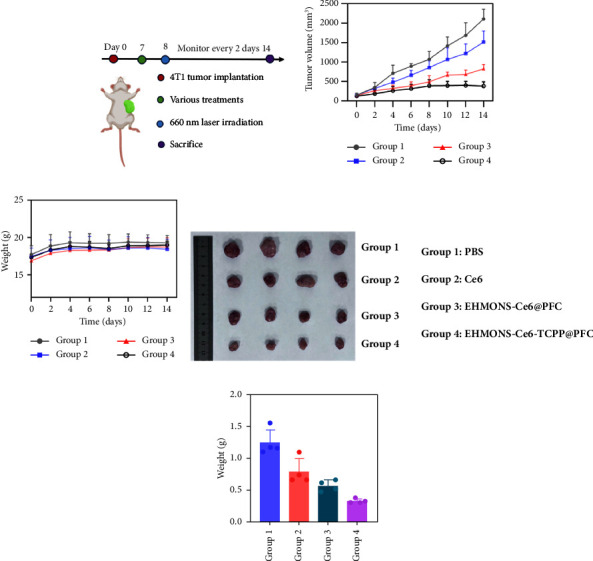
(a) Schematic illustration of the treatment protocol in 4TI breast tumor modal. (b) The tumor growth curves and (c) the body weight of mice in each group (group 1: PBS, group 2: Ce6, group 3: EHMONS-Ce6@PFC, group 4: EHMONS-Ce6-TCPP@PFC). (d, e) The photograph and the weight of tumors after different treatments (*n* = 4).

We further evaluated the in vivo anticancer efficacy of the mitochondria-targeting and oxygen-self-supplying nanomedicines for the breast cancer. To this end, the 4T1 tumor model was constructed by inoculation of 4T1 cells into BALB/c mice. On day 7, the 24 mice were randomly divided into 4 groups, and intratumorally administered with PBS, Ce6, EHMONs-Ce6@PFC, and EHMONs-Ce6-CTPP@PFC, followed by NIR irradiation at 24 h postadministration. The tumor volume of the mice was measured over 14 days (Figure 7(a)). The tumor volume of the PBS group with laser irradiation presented a remarkable increase and reached 1980 mm^3^ on day 14 (Figure 7(b)). By contrast, the free Ce6 exhibited a slight inhibition of tumor growth (1504 mm^3^) (Figure 7(b)). Moreover, the EHMONs-Ce6@PFC treatments showed moderate delay growth, while EHMONs-Ce6-CTPP@PFC treatment exhibits the distinct inhibition of tumor progression (392 mm^3^). This result indicated that the tumor growth could be effectively suppressed by combination of PFC and the mitochondria-targeting molecular TCPP. In addition, the administration of EHMONs-Ce6@PFC and EHMONs-Ce6-CTPP@PFC did not result in any significant reduction in body weight compared with the control groups (Figure 7(c)), indicating a lack of acute toxicity of the mitochondria-targeting and oxygen self-supplying nanomedicine. After the treatments, it was observed that the photographs and weights of tumor on the tumor-bearing mice exhibited the same results (Figures 7(d) and 7(e)), suggesting that the mitochondria-targeting and oxygen self-supplying EHMONs are a promising nanomedicine for the photodynamic therapy of tumors.

## 4. Conclusion

In summary, we construct an eccentric hollow nanoplatform with hypoxia-alleviating and mitochondrial-targeting ability for enhancing photodynamic efficacy. The prepared EHMONs possess a unique eccentric hollow structure, a large cavity, and ordered mesoporous channels. Thanks to their active surface properties and large cavity, the EHMONs are modified with the mitochondria-targeting molecule CTPP and photosensitizer Ce6 and loaded with oxygen-carrying compound PFCs. Hemolytic assays and *in vitro* toxicity experiments show that the EHMONs-Ce6-CTPP possesses good biocompatibility and mitochondrial targeting property, which increase the enrichment of photosensitizer Ce6 in mitochondria of tumor cells. The EHMONs-Ce6-CTPP@PFCs increase the content of singlet oxygen in 4T1 triple-negative breast cancer cells. Thus, the EHMONs-Ce6-CTPP@PFCs show a significantly improved killing effect for triple-negative breast cancer cells, which also verified in 4T1 tumor-bearing mouse models. This work provides a new route for photodynamic therapy of cancer by alleviating hypoxia and targeting mitochondrial strategies.

## Data Availability

The data supporting the findings of this study are available within the article.
